# Integrative score based on CDK6, PD-L1 and TMB predicts response to platinum-based chemotherapy and PD-1/PD-L1 blockade in muscle-invasive bladder cancer

**DOI:** 10.1038/s41416-023-02572-9

**Published:** 2024-01-11

**Authors:** Xiaohe Su, Kaifeng Jin, Qiji Guo, Ziyue Xu, Zhaopei Liu, Han Zeng, Yiwei Wang, Yu Zhu, Le Xu, Zewei Wang, Yuan Chang, Jiejie Xu

**Affiliations:** 1 NHC Key Laboratory of Glycoconjugate Research, Department of Biochemistry and Molecular Biology, School of Basic Medical Sciences, Fudan University, Shanghai, China; 2 Department of Urology, Zhongshan Hospital, Fudan University, Shanghai, China; 3 Department of Urology, Fudan University Shanghai Cancer Center, Shanghai, China; 4 Department of Urology, Shanghai Ninth People’s Hospital, Shanghai Jiao Tong University School of Medicine, Shanghai, China; 5 Department of Urology, Ruijin Hospital, Shanghai Jiao Tong University School of Medicine, Shanghai, China

**Keywords:** Bladder cancer, Cancer microenvironment, Cancer immunotherapy

## Abstract

**Background:**

Cyclin-dependent kinase 6 (CDK6) was proved to be an important regulator in the progression of cell cycle and has been a promising therapeutic target in cancer treatment. However, the clinical significance of CDK6 in muscle-invasive bladder cancer (MIBC) remains obscure. Herein, we attempt to explore the clinical relevance of CDK6 and assess the feasibility of the integrative model to predict immune checkpoint blockade (ICB) response.

**Methods:**

This study enrolled 933 patients with muscle-invasive bladder cancer (MIBC) from Zhongshan Hospital (ZSHS), The Cancer Genome Atlas (TCGA), Chemo, IMvigor210 and UC-GENOME cohorts. Kaplan-Meier survival and Cox regression analyses were performed to assess clinical outcomes based on CDK6 expression.

**Results:**

High CDK6 expression conferred poor prognosis and superior response to platinum-based chemotherapy but inferior response to ICB in MIBC. Furthermore, the integrative model named response score based on CDK6, PD-L1 and TMB could better predict the response to ICB and chemotherapy. Patients with higher response scores were characterised by inflamed immune microenvironment and genomic instability.

**Conclusions:**

CDK6 expression was correlated with prognosis and therapy response in MIBC. Integration of CDK6, PD-L1 and TMB could better identify patients who were most likely to benefit from ICB and chemotherapy.

## Introduction

Muscle-invasive bladder cancer (MIBC), which accounts for approximately 30% of all bladder cancer cases, is characterised by a high propensity to metastasise and high mortality [1]. For patients with MIBC, neoadjuvant or adjuvant chemotherapy (ACT) and immune checkpoint blockade (ICB) are recommended as the first-line treatments [2]. However, both chemotherapy and ICB exhibit limited clinical benefits due to suboptimal response rates and unavoidable adverse effects [3–5]. Therefore, clinicians tend to use predictive biomarkers to identify patients who are most likely to derive benefit from chemotherapy and ICB.

Up to now, no biomarkers have been approved to predict chemotherapy response in MIBC, although descriptive genomic analysis of pre-chemotherapy tumour samples has identified multiple genomic-associated biomarkers for chemotherapy response [6–8], which require further assessment in prospective clinical trials. For ICB, programmed cell death-ligand 1 (PD-L1) expression on tumour or immune cells and tumour mutation burden (TMB) were approved by the US Food and Drug Administration (FDA) to predict ICB response in diverse tumour types [9, 10]. However, PD-L1 or TMB alone was insufficient to identify all responders and non-responders [4, 11]. Therefore, it is of great clinical significance to investigate robust biomarkers of chemotherapy and ICB efficacy to select optimal treatment for each patient.

The cyclin-dependent kinase 6 (CDK6), in complex with cyclin D, regulates the progression of the cell cycle from G1 phase to S phase by phosphorylating the RB protein, which is critical for tumour cell survival and growth. Furthermore, recent studies have uncovered the impact of CDK6 on genomic stability and tumour immune microenvironment by regulating genes involved in the DNA replication and repair process and affecting the decisions of T cell fate [12, 13]. Therefore, CDK6 and its close homologue CDK4 have been viewed as promising therapeutic targets. Indeed, multiple CDK4/6 inhibitors have been approved to treat patients with hormone receptor-positive (HR^+^) breast cancer [14, 15]. However, the clinical significance of CDK6 in MIBC remains obscure.

To address the unmet needs mentioned above, we investigated the predictive valve of CDK6 on prognosis and response to chemotherapy and ICB in MIBC. Moreover, we proposed a ‘response score’ incorporating CDK6, PD-L1, and TMB to predict the response to ICB and characterised its correlation with immunogenic and genomic features in MIBC.

## Methods

### Study cohort

This study enrolled a total of 933 patients with MIBC from five independent cohorts, which was summarised in Supplementary Fig. 1.

For TCGA cohort, the clinical and genomic information of 412 patients diagnosed with bladder cancer was acquired via TCGA-Assembler 2.0.6 in July 2021. Based on the following Inclusion criteria, (i) data integrity of mRNA expression (*n* = 408) and overall survival (OS) information (*n* = 405); (ii) without neoadjuvant chemotherapy (*n* = 395); (iii) pathologically diagnosed as MIBC (*n* = 391), 391 cases were included ultimately. For ZSHS cohort, with the approval of the Clinical Research Ethics Committee of Zhongshan Hospital affiliated to Fudan University, 215 patients who received radical cystectomy at Zhongshan Hospital from 2002 to 2014 were followed up regularly till July 2016. With the following inclusion criteria, (i) pathologically confirmed as urothelial MIBC (*n* = 142); (ii) without dot loss in Formalin-fixed paraffin-embedded tumour microarray (TMA) (*n* = 114), 114 cases were enrolled in this study ultimately. For Chemo-cohort, which consisted of 149 patients treated with preoperative chemotherapy, the clinical and RNA-seq data were obtained from 10.1016/j.eururo.2021.10.035. Ultimately, 125 cases with MIBC receiving neoadjuvant chemotherapy were included, while 24 cases receiving induction chemotherapy were excluded.

For IMvigor210 cohort, which consisted of 348 patients treated with PD-L1 inhibitor atezolizumab in the IMvigor210 clinical trial, the clinical and RNA-seq data were obtained through ‘IMvigor210CoreBiologies’ R package from http://research-pub.gene.com/IMvigor210CoreBiologies. Ultimately, 195 patients with bladder derived urothelial carcinoma were included. For UC-GENOME cohort, the clinical, transcriptomic and genomic data of 218 patients diagnosed with metastatic urothelial carcinoma was acquired from cBioPortal for Cancer Genomics. With the following Inclusion criteria: data integrity of CDK6 sequencing data, PD-L1 sequencing data, TMB or survival data (*n* = 108), 108 cases were included ultimately. Detailed clinical and pathological characteristics of patients from the five cohorts were illustrated in Supplementary Table 1–6.

### RNA-seq data and data processing

The RNA-seq data of TCGA and IMvigor210 cohorts were obtained along with the process of acquiring clinical information. The transcript-related features for each specimen were converted to Fragments Per Kilobase of transcript per Million mapped reads (FPKM), and mRNA expression data was normalised by the formula log_2_(FPKM+1). The immune components of each tumour from TCGA and IMvigor210 cohorts and the immune-related signatures involved in this study were calculated as the average of normalised values of related genes as previously reported (genes were listed in Supplementary Table 7). The Immune score was calculated via ESTIMATE algorithm [16], which estimates the abundance of stromal and immune cells in tumour tissues based on gene expression data. Tumours from the TCGA cohort were categorised into four distinct tumour microenvironment subtypes (TME) based on functional gene expression signature scores, including immune-enriched, fibrotic (IF/F); immune-enriched, nonfibrotic (IE); fibrotic (F); and depleted (D) [17]. The molecular subtype of MIBC patients was estimated through BLCAsubtyping package from https://github.com/citbioinfo/BLCAsubtyping.

### Genomic analysis and variant assessment

For genomic analysis, TMB was identified as the number of somatic synonymous and non-synonymous mutations (base substitutions and indels) per megabase of analysed DNA. TMB in TCGA cohort was calculated by whole-exome sequencing (WES) while TMB in IMvigor210 trial UC-GENOME cohort was obtained by targeted gene sequencing. For further evaluation, TMB ≥ 10 mut/Mb was defined as ‘TMB high’, which was approved by the US FDA to predict salutary effects from ICB regardless of the tumour origins [10]. For mutational signatures, we estimated the contributions of different mutational signatures (COSMIC v.2, https://cancer.sanger.ac.uk/cosmic/signatures_v2) for each sample in TCGA cohort via SigProfiler (https://www.mathworks.com/matlabcentral/fileexchange/38724/). Besides, we summed the mutational signatures associated with same aetiology-APOBEC (signatures 2 and 13), defective homologous recombination repair (signature 3 and 8), tobacco smoking (signature 4), POLE (signature 10), defective DNA mismatch repair (signature 6, 14, 15, 20, 21 and 26), aging (signature 1 and 5), alkylating and ultraviolet (UV) (signature 7). Except above signatures, the left signatures were summed into other. In UC-GENOME cohort, the R package SomaticSignatures was used to identify mutational signatures (COSMIC v.3). Gene alterations were identified as either nonsense, missense, frameshift, splice-site, in frame deletion variants or deleterious homozygous deletions and amplifications. The data of copy-number variants in TCGA cohort originated from http://www.cbioportal.org. The data of APOBEC mutation load and neoantigen load of TCGA cohort was downloaded from previous study [18]. Simultaneously, the OncoKB dataset (https://www.oncokb.org/) was also matched with the TCGA cohort to delineate novel therapeutic targets.

### Assay methods

Immunohistochemistry (IHC) staining was performed on formalin-fixed, paraffin-embedded TMA in ZSHS cohort as described previously [19, 20]. Antibodies used for IHC staining were summarised in Supplementary Table 8. To evaluate the density of CDK6 expression, two independent pathologists who were blinded to clinicopathological data scored the samples separately. To evaluate the expression level of CDK6 protein, the IHC score of cytoplasmic staining was calculated by multiplication of intensity (stratified as negative (0), low (1), moderate (2), and high (3)) and the percentage of positive cells (0.0–1.0), which finally generated an IHC score ranging from 0 to 3 [21]. IHC score for each patient was evaluated as the average of 3 representative fields (200× magnification). The median values of CDK6 IHC score in ZSHS cohort and normalised CDK6 mRNA expression in TCGA cohort, Chemo-cohort, IMvigor210 cohort and UC-GENOME cohort were utilised as cut-off value for further analyses, which were 2.90 (IHC score), 1.675, 5.65, 0.715 and 0.68 respectively.

### Construction of the response score

In IMvgior210 cohort, PD-L1 expression on tumour-infiltrating immune cells (IC) was evaluated and proved to be a biomarker of ICB response [4]. Scoring criteria designated tumours as IC0, IC1, IC2, or IC3 if <1%, ≥1% but <5%, ≥5% but <10%, or ≥10% of IC were PD-L1 positive, respectively. This assay was validated for investigational use in clinical trials at the IC1 and IC2 cut-off. Since the data of PD-L1 expressing on tumour-infiltrating immune cells was not available in TCGA database and UC-GENOME cohort, we defined the top 50% as high PD-L1 mRNA expression. The cut-off points of PD-L1 mRNA expression in TCGA cohort and UC-GENOME cohort were 0.9196 FPKM and −0.07 (z-score normalised mRNA expression). Consistently, the top 50% was defined as high CDK6 expression in all cohorts involved and the cut-off points of CDK6 were mentioned as above. The response score was calculated by integrating three factors (CDK6 expression, PD-L1 expression and TMB), which stratified patients into four groups. Score one point for each criterion, including low CDK6 expression, PD-L1 expression on ≥5% of tumour-infiltrating immune cells (IC2+) or high PD-L1 expression and TMB ≥ 10 mut/Mb.

### Statistical analysis

The overall survival was determined by Kaplan-Meier analyses, which was evaluated by log-rank test. Multivariate and univariate analyses were performed to evaluate hazard ratio (HR, 95% confidence interval [CI]). The chi-squared test and Mann-Whitney test were applied in this study. Two-sided *P* < 0.05 was considered as statistically significant variation. All the statistical methods mentioned above were conducted through IBM SPSS Statistics 25.0 and R software 4.0.5.

## Results

### The predictive value of CDK6 on prognosis and therapeutic response in patients with MIBC

**Fig. 1:**
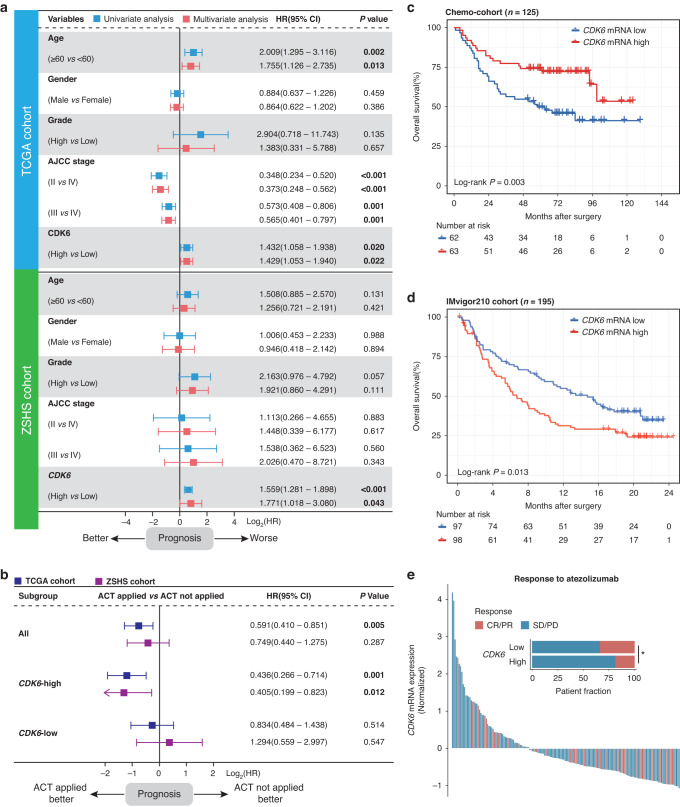
The predictive value of CDK6 on prognosis and therapeutic response in patients with MIBC. **a** Univariate and Multivariate Cox regression analysis of overall survival (OS) based on CDK6 expression in TCGA cohort (*n* = 391) and ZS cohort (*n* = 114). Due to the limited number of patients at AJCC stage IV in ZSHS cohort, the cox regression outcomes of AJCC stage were not accurate enough. **b** Cox regression analysis of overall survival (OS) based on ACT status in three subgroups (all patients, CDK6-high patients, CDK6-low patients) of TCGA cohort and ZS cohort. **c** Kaplan-Meier curves of overall survival (OS) based on CDK6 expression (HR: 0.439; 95% CI: 0.251–0.766) in Neo-cohort (*n* = 125). **d** Kaplan-Meier curve of overall survival (OS) based on CDK6 expression (HR: 1.551; 95% CI: 1.094–2.200) in IMvigor210 cohort (*n* = 195). **e** Waterfall plot and stacked bar plot demonstrated responsiveness to atezolizumab based on CDK6 expression in IMvigor210 cohort (*n* = 168). Data were analysed by Pearson’s chi-square test. Log-rank test was applied for Kaplan-Meier curves. All reported *p* values were two-sided. HR hazard ratio, CI confidence interval, ACT adjuvant chemotherapy, CR complete response, PR partial response, SD stable disease, PD progressive disease.

As the fundamental driver of the cell cycle, CDK6 is required for the initiation and progression of various malignancies [22, 23]. Therefore, we investigated the clinical significance of CDK6 expression in MIBC. The typical immunostaining image of CDK6 expression was displayed in Supplementary Fig. 2. To decipher the impact of CDK6 expression on the prognosis of patients with MIBC, Univariate and multivariate analysis were applied and demonstrated that CDK6 expression was an independent adverse prognosticator for OS of patients with MIBC in TCGA and ZSHS cohorts (Fig. 1a). Besides, we further examined the impact of CDK6 expression on recurrence-free survival (RFS) of patients with MIBC. Consistent with OS, univariate and multivariate analysis demonstrated that CDK6 expression was also an independent adverse prognosticator for RFS of patients with MIBC in both TCGA and ZSHS cohorts (Supplementary Fig. 3).

Since most drugs used for standard cytotoxic chemotherapy target the tumour cells during active proliferation, we hypothesised that patients with high levels of CDK6 expression may be more likely to benefit from chemotherapy. We further investigated the relationship between CDK6 expression and survival benefit from ACT in TCGA and ZS cohorts and neoadjuvant chemotherapy in Chemo-cohort. The application of ACT provided significant survival benefit for patients with MIBC in TCGA cohort, while the patients in ZS cohort could not derive survival benefit from ACT (Fig. 1b). For patients stratified by the levels of CDK6 expression, survival benefit from ACT was only observed in CDK6-high subgroup in TCGA and ZSHS cohorts (Fig. 1b). To further verify above results, Chemo-cohort in which patients received neoadjuvant chemotherapy was enrolled. The result showed that patients with high CDK6 expression had significantly better OS from neoadjuvant chemotherapy as well. (Fig. 1c). Above results suggested that CDK6 expression might serve as a potential predictive marker of chemotherapy response for patients with MIBC.

In addition to the impact on chemotherapy response, CDK6 has been shown as a master regulator of the immune resistance programme in melanoma and inhibition of CDK6 represses the resistance programme and improves responses to ICB in vivo [24]. To evaluate the predictive value of CDK6 expression for ICB response in MIBC, IMvigor210 cohort consisting of patients treated with atezolizumab was involved in this study. The result demonstrated that patients in CDK6-high subgroup exhibited a significantly worse OS compared with those in CDK6-low subgroup (Fig. 1d). Meanwhile, patients in CDK6-high subgroup showed a significantly decreased clinical response rate, suggesting an association between CDK6 expression and resistance to PD-L1 blockade (Fig. 1e).

### The predictive value of response score incorporating CDK6, PD-L1 and TMB on response to ICB in MIBC

**Fig. 2:**
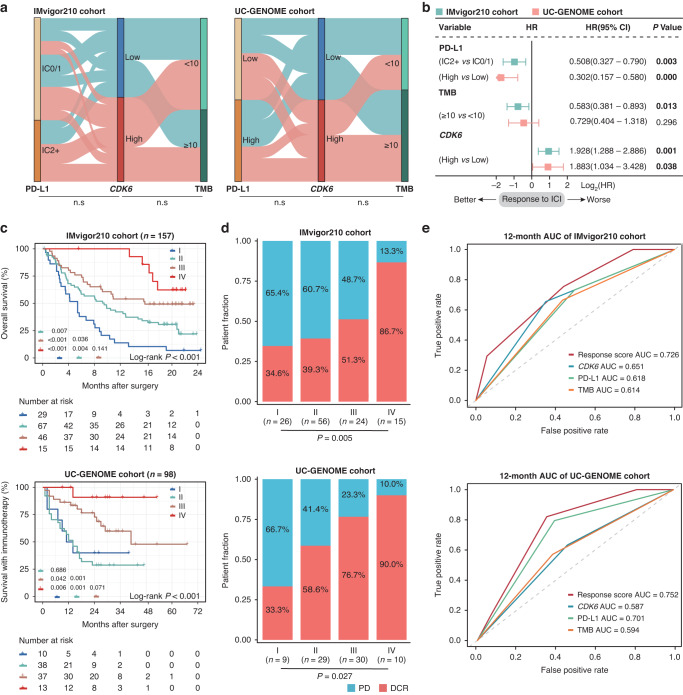
The predictive value of response score incorporating CDK6, PD-L1 and TMB on ICI in MIBC. **a** Sankey diagram displaying the relationship between TMB, PD-L1 and CDK6 in IMvigor210 cohort and UC-GENOME cohort. **b** Multivariate Cox regression analysis was conducted based on PD-L1, TMB and CDK6 in IMvigor210 cohort and UC-GENOME cohort. **c** Kaplan-Meier curve of overall survival (OS) based on response score in IMvigor210 cohort (*n* = 157) (I: HR: 6.888; 95% CI: 2.631–18.034; II: HR: 3.536; 95% CI: 1.401–8.924; III: HR: 2.053; 95% CI: 0.780–5.404) and UC-GENOME cohort (*n* = 98) (I: HR: 12.826; 95% CI: 1.539–106.912; II: HR: 14.744; 95% CI: 1.996–108.892; III: HR: 5.238; 95% CI: 0.685–40.049). **d** Stacked bar plot demonstrated response to ICI based on response score. **e** Time-dependent ROC analysis for response score, CDK6, PD-L1 and TMB in IMvigor210 cohort and UC-GENOME cohort. Chi-square test was applied. Log-rank test was applied for Kaplan-Meier curves. All reported *p*-values were two-sided. Ns referred to not statistically significant. PD-L1 programmed cell death-ligand 1, TMB tumour mutation burden, IC immune cell, PD progressive disease, DCR disease control rate, AUC area under curve.

Given the predictive ability of acknowledged biomarkers such as PD-L1 and TMB [25], we further assessed the correlation between CDK6 and PD-L1, TMB in IMvigor210 cohort and UC-GENOME cohort in which patients received ICB. Given the absence of data on PD-L1 expression level on immune cells (IC) by IHC in UC-GENOME cohort, we employed CD274 (PD-L1) mRNA expression as an alternative approach. To ensure its feasibility, we further investigated the association of PD-L1 expression on IC and mRNA expression in IMvigor210 cohort. The result demonstrated that CD274 (PD-L1) mRNA expression of IC2+ patients was significantly increased than IC0/1 patients (Supplementary Fig. 4), which indicated a high correlation between PD-L1 expression on IC and mRNA expression. Notably, there was no significant correlation of CDK6 stratification with PD-L1 expression level and TMB in both cohorts (Fig. 2a). Furthermore, multivariable cox regression analysis showed that CDK6 expression could serve as a strong prognosticator of shorter survival in patients who received ICB, independent of PD-L1 and TMB levels, which suggested CDK6 might be a relatively independent biomarker of ICB response (Fig. 2b).

Although established biomarkers such as PD-L1 and TMB showed some predictive value, each biomarker alone missed important subgroups of patients who could potentially benefit from ICB [4, 26]. As an independent candidate biomarker for ICB response, we wondered whether CDK6 expression could define more potential ICB-sensitive patients combined with PD-L1 and TMB. Thus, we built a ‘response score’ incorporating CDK6, PD-L1, TMB and stratified patients into four groups (I to IV with 0 to 3 response score). Remarkably, patients with higher response scores showed superior OS after treatment with ICB in both IMvigor210 cohort and UC-GENOME cohort (Fig. 2c). Besides, patients with the highest response score had the highest disease control rate (DCR) in both cohorts (Fig. 2d). Remarkably, the response score showed better predictive value of ICB response than the pairwise combination of CDK6, PD-L1 and TMB in both cohorts, which showed improved but unsatisfactory performance in definition to ICI-sensitive patients (Supplementary Fig. 5A–F). Consistent with above results, the areas under curve of response score for 12-month OS were 0.726 and 0.752 in IMvigor210 cohort and UC-GENOME cohort respectively, which showed superiority over CDK6, PD-L1 or TMB alone (Fig. 2e). In summary, we demonstrated that the integrated model could more accurately define ICB-sensitive patients.

### The predictive value of response score incorporating CDK6, PD-L1 and TMB on chemotherapy response in MIBC

The results had demonstrated the convincing predictive ability of response score for ICB response. We further explored whether the response score was associated with chemotherapy response in TCGA cohort and UC-GENOME cohort. Notably, patients with highest response scores showed the best OS in TCGA cohort and best survival with chemotherapy in UC-GENOME cohort, although there was no statistically significant difference across response score subgroups in UC-GENOME cohort (Supplementary Fig. 6A, B).

### Immunogenic features of MIBC stratified by response score

**Fig. 3:**
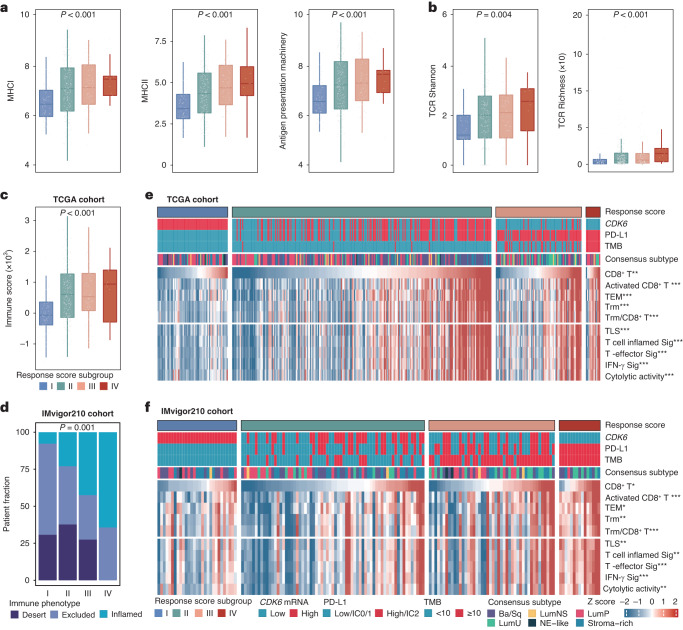
Immunogenic features of MIBC patients stratified by CDK6 expression. **a** Box plots for the signature of MHC-I, MHC-II and antigen presentation machinery (APM) in TCGA cohort. **b** Violin plots illustrating the differences in T cell receptor (TCR) diversity (TCR Shannon) and richness within response score subgroups in TCGA cohort. **c** Box plot for immune score in TCGA cohort. **d** Stacked bar chart illustrating the distribution of the immune phenotypes in response score subgroups in IMvigor210 cohort. Heatmap for the correlation between response score and immune indicators in TCGA cohort (**e**) and IMvigor210 cohort (**f**). The mean rank of Kruskal-Wallis test was standardised by z-score in Fig. 3f. Kruskal-Wallis test and Chi-square test were applied. All reported *p* values were two sided. *P < 0.05, **p < 0.01 and ***p < 0.001. MHC major histocompatibility complex, TCR T cell receptor, TEM effector memory T cells, Trm tissue resident memory T cells.

To better elucidate the predictive value of response score for therapy response, we further investigated the difference in immunogenic features among patients with MIBC stratified by response score. The results showed that signalling pathways related to MHC I, MHC II and antigen presentation machinery were significantly upregulated in patients with higher response scores in TCGA cohort (Fig. 3a). The same results were also seen in IMvigor210 cohort and UC-GENOME cohort (Supplementary Fig. 7A, B). Consistently, T cell receptor (TCR) diversity and richness were significantly increased in patients with higher response scores (Fig. 3b). Furthermore, we investigated characteristics of the downstream immune components. Higher immune scores were observed in patients with higher response scores from TCGA cohort while inflamed immune phenotype was enriched in patients with higher response scores from IMvigor210 cohort (Fig. 3d). Besides, we categorised tumours from the TCGA cohort into four distinct tumour microenvironment (TME) subtypes based on functional gene expression signature scores, including IF/F; IE; F; and D [17]. Consistent with the result in IMvigor210 cohort, patients with the highest response score mainly exhibited an IE TME subtype in TCGA cohort (Supplementary Fig. 8). Moreover, we found that the immune-activated cells (CD8^+^ T cells, activated CD8^+^ T cells, effector memory T cells (TEM), tissue-resident memory T cells (Trm) and Trm ratio to CD8^+^ T cells) and immune-related signatures (tertiary lymphoid structure (TLS), T cell inflamed, T effector, interferon-gamma and cytolytic activity) were enriched in patients with higher response scores from TCGA cohort and IMvigor210 cohort (Fig. 3e, f). All of the above results suggested the tumours with higher response scores tended to possess a more favourable inflamed and anti-tumour immune microenvironment.

### Genomic landscape of MIBC stratified by response score

**Fig. 4:**
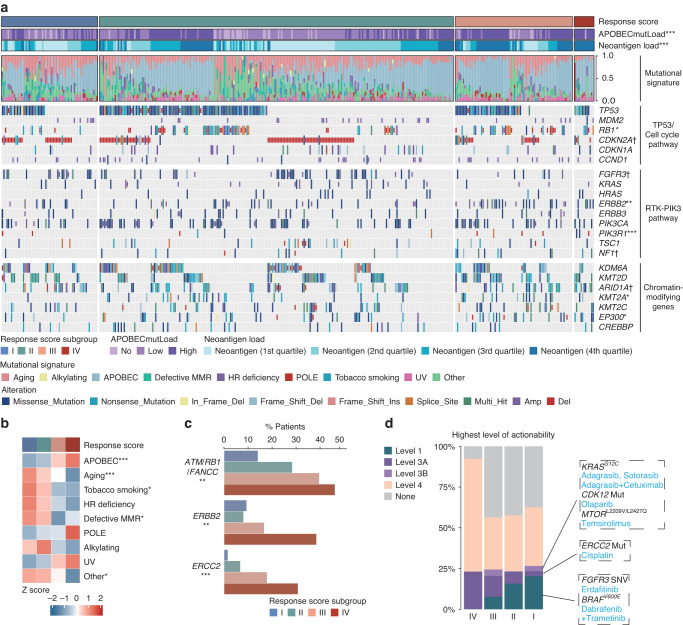
Genomic landscape of MIBC stratified by response score. **a** Alteration landscape of MIBC stratified by response score in TCGA cohort. Top to bottom: APOBEC mutation load, neoantigen load by quartile, mutational signatures and alteration of genes from TP53/cell cycle pathway, RTK-PIK3 pathway and chromatin-modifying pathway. **b** Heatmap for mutational signatures. **c** Prevalence of gene alterations associated with chemotherapy responsiveness in MIBC with response score. **d** Highest levels of therapeutic actionability in MIBC stratified by response score. The mean rank of Kruskal-Wallis test was standardised by z-score in Fig. 3f. Kruskal-Wallis test, Chi-square test and Fisher’s exact test were applied. All reported *p*-values were two-sided. ^†^*P* < 0.1, *p < 0.05, **p < 0.01 and ***p < 0.001. MMR mismatch repair, HR homologous recombination, UV ultraviolet.

As upstream events in the TME, genomic alterations are sources for the crosstalk between the tumour and its immune microenvironment. Thus, we further investigated the genomic discrepancy of MIBC stratified by response score. At first, we examined the correlation between response score and mutational signatures denoting genomic instability in TCGA cohort and UC-GENOME cohort. APOBEC mutational signatures were significantly enriched in patients with higher response scores in both cohorts (Fig. 4a, b; Supplementary Fig. 9B). Consistently, APOBEC mutation load was also elevated in patients with higher response scores in TCGA cohort (Fig. 4a), which was reported to generate more neoepitopes in tumour cell vaccines [27]. Consistently, neoantigen load was significantly increased in patients with higher response scores in TCGA cohort and IMvigor210 cohort (Fig. 4a; Supplementary Fig. 9A). Besides, we assessed the association between response score and deficiency in TP53/cell cycle pathway, RTK-PIK3 pathway and chromatin-modifying genes in TCGA cohort and IMvigor210 cohort. Notably, recurrent alterations of genes from pathways mentioned above were observed in patients with higher response scores in both cohorts, including RB1, PIK3R1, ARID1A (Fig. 4a; Supplementary Fig. 9C). Emphatically, we tested the correlation of response score with somatic mutations in ATM/RB1/FANCC, ERBB2 and ERCC2, which have been previously shown to predict response to neoadjuvant cisplatin-based chemotherapy [6–8]. In accordance with our findings above, more recurrent mutations of ATM/RB1/FANCC, ERBB2 and ERCC2 were observed in patients with higher response scores (Fig. 4c). Finally, we utilised the OncoKB dataset, which embodies a comprehensive and curated precision oncology knowledge base and offers oncologists detailed, evidence-based information about individual somatic mutations and structural alterations, to investigate additional therapy choices for patients stratified by response score. The results showed that patients with lowest response scores tended to harbour FGFR3 single nucleotide variants (SNV) and BRAF^V600E^, which was categorised as level 1 of actionability, and thus might benefit from FGFR3 inhibitors and BRAF^V600E^ inhibitors (Fig. 4d).

## Discussion

Evading growth suppression and unlimited cell proliferation is one of the hallmarks of cancer [28]. As the fundamental driver of the cell cycle from G1 phase to S phase, CDK6 plays an important role in the progression of cancer [22, 23]. However, the clinical significance of CDK6 in MIBC is not fully characterised. In this study, we revealed that MIBC patients with high levels of CDK6 expression exhibited inferior OS independent of other pathological factors but better response to platinum-based chemotherapy. For ICB, high levels of CDK6 expression were associated with immune resistance to ICB. Notably, we found that there was no significant correlation of CDK6 expression with PD-L1 expression and TMB, which suggested that the impact of CDK6 on ICB response was independent of PD-L1 expression and TMB. CDK6 could serve as a complementary predictor of ICB response for PD-L1 expression and TMB.

ICB, which reactivates intratumour T cells via the axis of PD-L1/PD-1, has shown promising results in selected patients with bladder cancer [4, 29]. However, ICB benefits only a subset of patients, and a substantial proportion of these individuals eventually manifest resistance. Additionally, serious immune-related adverse events have been reported in a clinically significant proportion of patients who receive ICB [5]. Currently, multiple biomarkers have been identified to predict response to ICB in MIBC, including PD-L1 expression on immune cells and TMB. However, there is still a subset of patients with PD-L1-negative and/or TMB-low tumours responding to ICB and another subset of patients with both PD-L1-positive and TMB-high tumours manifesting resistance to ICB [4, 26]. Therefore, whether PD-L1 expression or TMB as a solitary biomarker for ICB benefit is suboptimal, which highlights the need to identify new biomarkers and develop an integrated predictive model of ICB response. In our study, we proposed an integrated predictive model named ‘response score’, which incorporated CDK6, PD-L1 and TMB and demonstrated that response score outperformed CDK6, PD-L1 or TMB alone and their pairwise combination in identifying patients who were most likely to benefit from ICB. When applying the integrated model in predicting chemotherapy response, response score also exhibited an outstanding performance in identifying patients who were most likely to derive survival benefits from chemotherapy. Moreover, high-dimensional integration of the genomic and immunogenic data allowed us to elucidate the potential mechanisms by which the response score impacted the efficacy of ICB and chemotherapy.

For tumour immune microenvironment, previous studies have shown its close association with the efficacy of ICB and chemotherapy, especially the status of immune microenvironment [30], which was also reported in our recent studies [19, 20, 31]. Given the evidence that uninflamed cold tumours are unlikely to respond to ICB and inflamed hot tumours are more likely to benefit from ICB [4, 17], we further examined the immunogenic features shaped by the response score. Enhanced antigen presentation and enriched TCR, indicating the magnitude of immune reaction, were observed in patients with higher response scores. Consistently, we observed increased infiltration of immune cells (e.g., CD8^+^ T cells, TEM and Trm) and upregulation of immune-related signatures (e.g., TLS, T cell inflamed and T effector). Taken together, patients with higher response scores were more likely to exhibit inflamed immune phenotypes, which were most likely to derive benefit from ICB.

With the development of gene sequencing technology in the past few decades, genomic profiling is becoming a routine component for patients to predict clinical outcomes and responses to therapies in diverse tumour types [18, 32, 33]. For MIBC, recent studies have reported the association between genetic alterations and response to chemotherapy and ICB [6–8, 34, 35]. Therefore, we assessed the genomic features of MIBC stratified by response score. Patients with higher response scores were characterised by enriched APOBEC mutational signatures and neoantigens, which have been reported to be predictors of improved ICB efficacy [27, 36]. Moreover, we summarised the genes from TP53/cell cycle pathway, RTK-PIK3 pathway and chromatin-modifying pathway, which accounts for the highest mutation frequency in MIBC [18]. The data showed a trend towards a higher frequency of recurrent gene alterations (e.g., RB1, PIK3R1) in patients with higher response scores, which suggested intrinsic driving forces of genomic instability. Previous studies have reported that ATM/RB1/FANCC alterations, ERBB2 mutations and ERCC2 mutations correlate with sensitivity to platinum-based chemotherapy [6–8]. Consistently, patients with higher response scores were characterised by more recurrent mutations of ATM/RB1/FANCC, ERBB2 and ERCC2 and exhibited improved survival benefit from chemotherapy. In addition to predicting responses to existing therapies, genomic profiling also makes it possible to identify additional actionable therapeutic targets and provide patients with more therapy options, especially when exhibiting resistant to conventional therapies. To identify novel therapy options for patients with lowest response score, who were found to be resistant to both ICB and chemotherapy, we employed the OncoKB dataset and revealed that patients with lowest response score might benefit from FGFR3 inhibitors and BRAF^V600E^ inhibitors.

Several limitations of our study deserve attention. Although the predictive ability of CDK6 and response score incorporating CDK6, PD-L1 and TMB on prognosis and therapy response was proved across multiple cohorts we enrolled in MIBC, it was retrospective and further validation is necessary to confirm our findings through extensive, multi-centred clinical trials. Besides, assessment methods of PD-L1 expression and TMB differed among the cohorts included in our study. For example, IHC was used to assess PD-L1 expression level on immune cells, while RNA sequencing was applied in other cohorts. TMB was calculated based on the data of whole genome sequencing in TCGA cohort. But in other cohorts, it was calculated based on the data of next-generation targeted sequencing. Further studies are needed to investigate the impact of these assessment methods. At last, the thresholds of CDK6 and PD-L1 expression were not consistent, which required further validation.

In summary, our study demonstrated CDK6 was associated with adverse outcomes and contributed to chemotherapy sensitivity and ICB resistance in MIBC. Moreover, an integrated model of CDK6, PD-L1 and TMB was proposed and showed improved performance in the prediction of ICB and chemotherapy efficacy. Our study may inform the significance of CDK6 combined with PD-L1 and TMB as an integrated model for precise stratification and personalised therapy of patients with MIBC.

### Supplementary information

Supplementary Table

Supplementary Figures

## Data Availability

Data and materials generated that are relevant to the results are included in this article. Other data are available from the corresponding author Prof. Xu, upon reasonable request.
