# Supplementary material for: Integrative score based on CDK6, PD-L1 and TMB predicts response to platinum-based chemotherapy and PD-1/PD-L1 blockade in muscle-invasive bladder cancer
Source: Br J Cancer. 2024 Jan 11;130(5):852–60. doi: 10.1038/s41416-023-02572-9 (PMC10912081; doi:10.1038/s41416-023-02572-9)
Supplement: Supplementary file 1 — Supplementary Table [file 41416_2023_2572_MOESM1_ESM.docx]

**Supplementary Table 1. Association between CDK6 expression and clinicopathological parameters in TCGA cohort and ZS cohort.**

| Characteristics | ZS cohort (*n* = 114) | | | | |  | TCGA cohort (*n* = 391) | | | | |
| --- | --- | --- | --- | --- | --- | --- | --- | --- | --- | --- | --- |
|  | **Patients**  **No. %** | | **CDK6 expression**  **high** | **CDK6 expression**  **low** | ***P**** |  | **Patients**  **No. %** | | **CDK6 expression**  **high** | **CDK6 expression**  **low** | ***P**** |
| All patients | 114 | 100.0 | 57 | 57 |  |  | 391 | 100.0 | 196 | 195 |  |
| Age (year) |  |  |  |  | 0.058 |  |  |  |  |  | 0.313 |
| <60 | 48 | 42.11 | 19 | 29 |  |  | 84 | 21.48 | 38 | 46 |  |
| ≥60 | 66 | 57.89 | 38 | 28 |  |  | 307 | 78.52 | 158 | 149 |  |
| Gender |  |  |  |  | 0.189 |  |  |  |  |  | 0.671 |
| Male | 97 | 85.09 | 51 | 46 |  |  | 285 | 72.89 | 141 | 144 |  |
| Female | 17 | 14.91 | 6 | 11 |  |  | 106 | 27.11 | 55 | 51 |  |
| Grade |  |  |  |  | **0.024** |  |  |  |  |  | 0.061 |
| Low grade | 19 | 16.67 | 5 | 14 |  |  | 20 | 5.12 | 6 | 14 |  |
| High grade | 95 | 83.33 | 52 | 43 |  |  | 369 | 94.37 | 190 | 179 |  |
| pT stage |  |  |  |  | **0.014** |  |  |  |  |  | **0.009** |
| pT2 | 72 | 63.16 | 29 | 43 |  |  | 113 | 28.90 | 45 | 68 |  |
| pT3 | 26 | 22.81 | 19 | 7 |  |  | 189 | 48.34 | 109 | 80 |  |
| pT4 | 16 | 14.04 | 9 | 7 |  |  | 56 | 14.32 | 26 | 30 |  |
| pN stage |  |  |  |  | 0.647 |  |  |  |  |  | 0.154 |
| pN0 | 109 | 95.61 | 54 | 55 |  |  | 228 | 58.31 | 122 | 106 |  |
| pN+ | 5 | 4.39 | 3 | 2 |  |  | 123 | 31.46 | 56 | 67 |  |
| LVI |  |  |  |  | 0.082 |  |  |  |  |  | 0.589 |
| Absent | 43 | 37.72 | 17 | 26 |  |  | 125 | 31.97 | 64 | 61 |  |
| Present | 71 | 62.28 | 40 | 31 |  |  | 142 | 36.32 | 68 | 74 |  |
| AJCC stage |  |  |  |  | **0.034** |  |  |  |  |  | **0.016** |
| II | 69 | 60.53 | 28 | 41 |  |  | 125 | 31.97 | 54 | 71 |  |
| III | 40 | 35.09 | 25 | 15 |  |  | 137 | 35.04 | 82 | 55 |  |
| IV | 5 | 4.39 | 4 | 1 |  |  | 129 | 32.99 | 60 | 69 |  |
| ACT |  |  |  |  | 0.574 |  |  |  |  |  | 0.272 |
| Applied | 57 | 50.00 | 30 | 27 |  |  | 94 | 44.76 | 52 | 42 |  |
| Not applied | 57 | 50.00 | 27 | 30 |  |  | 293 | 18.16 | 143 | 150 |  |

**Abbreviations**: LVI: lymphatic vessel invasions; AJCC: American Joint Committee on Cancer; ACT: adjuvant chemotherapy.

**P* value was used from Chi-square test; significant *P* value < 0.05 was shown in bold.

**Supplementary Table 2. Association between CDK6 expression and clinicopathological parameters in Chemo-cohort.**

| Characteristics | Chemo-cohort (*n* = 125) | | | | |
| --- | --- | --- | --- | --- | --- |
|  | **Patients**  **No. %** | | **CDK6 expression**  **high** | **CDK6 expression**  **low** | ***P**** |
| All patients | 125 | 100.0 | 63 | 62 |  |
| Age (year) |  |  |  |  | 0.627 |
| <60 | 26 | 20.80 | 12 | 14 |  |
| ≥60 | 99 | 79.20 | 51 | 48 |  |
| Gender |  |  |  |  | 0.117 |
| Male | 98 | 78.40 | 53 | 45 |  |
| Female | 27 | 21.60 | 10 | 17 |  |
| pT stage |  |  |  |  | **0.047** |
| pT2 | 53 | 42.40 | 32 | 21 |  |
| pT3 | 62 | 49.60 | 29 | 33 |  |
| pT4 | 10 | 8.00 | 2 | 8 |  |

**Abbreviations**: LVI: lymphatic vessel invasions; AJCC: American Joint Committee on Cancer; ACT: adjuvant chemotherapy.

**P* value was used from Chi-square test; significant *P* value < 0.05 was shown in bold.

**Supplementary Table 3. Association between CDK6 expression and clinicopathological parameters in IMvigor210 cohort.**

| Characteristics | IMvigor210 cohort (*n* = 195) | | | | |
| --- | --- | --- | --- | --- | --- |
|  | **Patients**  **No. %** | | **CDK6 expression**  **high** | **CDK6 expression**  **low** | ***P**** |
| All patients | 195 | 100 | 98 | 97 |  |
| Gender |  |  |  |  | 0.756 |
| Male | 42 | 21.54 | 22 | 20 |  |
| Female | 153 | 78.46 | 76 | 77 |  |
| PD-L1+ IC Level |  |  |  |  | 0.086 |
| IC0/1 | 128 | 65.64 | 59 | 69 |  |
| IC2+ | 66 | 33.85 | 39 | 27 |  |
| TMB |  |  |  |  | 0.403 |
| High(≥10) | 69 | 35.38 | 33 | 36 |  |
| Low(＜10) | 88 | 45.13 | 48 | 40 |  |
| Response |  |  |  |  | **0.040** |
| CR/PR | 42 | 21.54 | 15 | 27 |  |
| SD/PD | 126 | 64.62 | 68 | 58 |  |

**Abbreviations**: IC: immune cell; TMB: tumour mutation burden; CR: complete response; PR: partial response; SD: stable disease; PD: progressive disease.

**P* value was used from Chi-square test; significant *P* value < 0.05 was shown in bold.

**Supplementary Table 4. Association between response score and clinicopathological parameters in IMvigor210 cohort.**

| Characteristics | IMvigor210 cohort (*n* = 157) | | | | | | |
| --- | --- | --- | --- | --- | --- | --- | --- |
|  | **Patients**  **No. %** | | **I** | **II** | **III** | **IV** | ***P**** |
| All patients | 157 | 100.0 | 29 | 67 | 46 | 15 |  |
| Gender |  |  |  |  |  |  | 0.223 |
| Male | 124 | 79.00 | 20 | 51 | 40 | 13 |  |
| Female | 33 | 21.00 | 9 | 16 | 6 | 2 |  |
| Immune phenotype |  |  |  |  |  |  | **0.001** |
| Desert | 42 | 26.75 | 8 | 23 | 11 | 0 |  |
| Excluded | 57 | 36.31 | 16 | 24 | 12 | 5 |  |
| Inflamed | 42 | 26.75 | 2 | 14 | 17 | 9 |  |
| Clinical benefit |  |  |  |  |  |  | **0.005** |
| DCR | 64 | 40.76 | 9 | 22 | 20 | 13 |  |
| PD | 72 | 45.86 | 17 | 34 | 19 | 2 |  |

**Abbreviations**: DCR: disease control rate; PD: progressive disease.

**P* value was used from Chi-square test; significant *P* value < 0.05 was shown in bold.

**Supplementary Table 5. Association between response score and clinicopathological parameters in UC-GENOME cohort.**

| Characteristics | UC-GENOME cohort (*n* = 108) | | | | | | |
| --- | --- | --- | --- | --- | --- | --- | --- |
|  | **Patients**  **No. %** | | **I** | **II** | **III** | **IV** | ***P**** |
| All patients | 108 | 100.0 | 11 | 41 | 41 | 15 |  |
| Gender |  |  |  |  |  |  | 0.658 |
| Male | 80 | 74.07 | 8 | 28 | 33 | 11 |  |
| Female | 28 | 25.93 | 3 | 13 | 8 | 4 |  |
| Age |  |  |  |  |  |  | 0.069 |
| <60 | 28 | 25.9 | 3 | 8 | 9 | 8 |  |
| ≥60 | 80 | 74.1 | 8 | 33 | 32 | 7 |  |
| Immune phenotype |  |  |  |  |  |  | **0.009** |
| Desert | 1 | 0.93 | 1 | 0 | 0 | 0 |  |
| Excluded | 58 | 53.70 | 7 | 26 | 18 | 7 |  |
| Inflamed | 41 | 37.96 | 1 | 12 | 21 | 7 |  |
| Clinical benefit |  |  |  |  |  |  | **0.027** |
| DCR | 52 | 48.15 | 3 | 17 | 23 | 9 |  |
| PD | 26 | 24.07 | 6 | 12 | 7 | 1 |  |

**Abbreviations**: DCR: disease control rate; PD: progressive disease.

**P* value was used from Chi-square test; significant *P* value < 0.05 was shown in bold.

**Supplementary Table 6. Association between response score and clinicopathological parameters in TCGA cohort.**

| Characteristics | TCGA cohort (*n* = 391) | | | | | | |
| --- | --- | --- | --- | --- | --- | --- | --- |
|  | **Patients**  **No. %** | | **I** | **II** | **III** | **IV** | ***P**** |
| All patients | 391 | 100.0 | 65 | 236 | 78 | 13 |  |
| Gender |  |  |  |  |  |  | 0.369 |
| Male | 285 | 72.89 | 50 | 165 | 61 | 9 |  |
| Female | 106 | 27.11 | 14 | 71 | 17 | 4 |  |
| Age |  |  |  |  |  |  | 0.995 |
| <60 | 84 | 21.48 | 14 | 51 | 16 | 3 |  |
| ≥60 | 307 | 78.52 | 50 | 185 | 62 | 10 |  |
| Grade |  |  |  |  |  |  | 0.230 |
| Low grade | 20 | 5.12 | 3 | 16 | 1 | 0 |  |
| High grade | 369 | 94.37 | 61 | 220 | 75 | 13 |  |
| pT stage |  |  |  |  |  |  | 0.260 |
| pT2 | 113 | 28.90 | 12 | 73 | 24 | 4 |  |
| pT3 | 189 | 48.34 | 36 | 114 | 33 | 6 |  |
| pT4 | 56 | 14.32 | 7 | 34 | 15 | 0 |  |
| pN stage |  |  |  |  |  |  | 0.684 |
| pN0 | 228 | 58.31 | 35 | 144 | 42 | 7 |  |
| pN+ | 123 | 31.46 | 21 | 73 | 27 | 2 |  |
| LVI |  |  |  |  |  |  | 0.088 |
| Absent | 125 | 32.00 | 13 | 75 | 31 | 6 |  |
| Present | 142 | 36.32 | 24 | 86 | 31 | 1 |  |
| AJCC stage |  |  |  |  |  |  | 0.844 |
| II | 125 | 32.00 | 18 | 77 | 24 | 6 |  |
| III | 137 | 35.04 | 23 | 82 | 27 | 5 |  |
| IV | 129 | 33.00 | 23 | 77 | 27 | 2 |  |
| ACT |  |  |  |  |  |  | 0.387 |
| Applied | 94 | 24.04 | 18 | 52 | 19 | 5 |  |
| Not applied | 293 | 74.94 | 46 | 182 | 58 | 7 |  |

**Abbreviations**: LVI: lymphatic vessel invasions; AJCC: American Joint Committee on Cancer; ACT: adjuvant chemotherapy.

**P* value was used from Chi-square test; significant *P* value < 0.05 was shown in bold.

**Supplementary Table 7. Specific gene signature list.**

| Signature | Gene | Source |
| --- | --- | --- |
| MHC-I | *HLA-A, HLA-B, HLA-C, B2M, TAP1, TAP2, TAPBP* | PMID: 34019806 |
| MHC-II | *HLA-DRA, HLA-DRB1, HLA-DMA, HLA-DPA1, HLA-DPB1, HLA-DMB, HLA-DQB1, HLA-DQA1, CIITA* | PMID: 34019806 |
| Antigen presentation machinery | *HLA-A, HLA-B, HLA-C, TAP1, TAP2* | PMID: 27855702 |
| CD8 T cell | *CD8A, CD8B, PF4, PRR5, SF1, LIME1, DNAJB1, ARHGAP8, GZMM, SLC16A7, SFRS7, APBA2, C4orf15, LEPROTL1, ZFP36L2, MYST3, ZEB1, ZNF609, C12orf47, THUMPD1, VAMP2, ZNF91, ZNF22, TMC6, DNAJB1, FLT3LG, CDKN2AIP, TSC22D3, TBCC, RBM3, ABT1, C19orf6, CAMLG, PPP1R2, AES, KLF9, PRF1* | PMID: 35970919 |
| Activated CD8 T cell | *ADRM1, AHSA1, C1GALT1C1, CCT6B, CD37, CD3D, CD3E, CD3G, CD69, CD8A, CETN3, CSE1L, GEMIN6, GNLY, GPT2, GZMA, GZMH, GZMK, IL2RB, LCK, MPZL1, NKG7, PIK3IP1, PTRH2, TIMM13, ZAP70* | PMID: 28052254 |
| Effector memory CD8 T cell | *ACAP1, APOL3, ARHGAP10, ATP10D, C3AR1, CCR5, CD160, CD55, CFLAR, CMKLR1, DAPP1, FCRL6, FLT3LG, GZMM, HAPLN3, HLA-DMB, HLA-DPA1, HLA-DPB1, IFI16, LIME1, LTK, NFKBIA, SETD7, SIK1, TRIB2* | PMID: 28052254 |
| Tissue-resident memory T cell | ITGAE, PDCD1, CD69, ITGA1, HAVCR2, GZMB, CTLA4, KIR2DL4, ENTPD1, LAG3, TNFSF4, TNFRSF9, IFNG, LAYN, FABP5, TIGIT, BAG3, GZMH, PRF1, FASLG, CD8A | PMID: 32112054 |
| Tertiary lymphoid structure | *CCL2, CCL3, CCL4, CCL5, CCL8, CCL18, CCL19, CCL21, CXCL9, CXCL10, CXCL11, CXCL13* | PMID: 23097687 |
| T cell-inflamed signature | *IRF1, CD8A, CCL2, CCL3, CCL4, CXCL9, CXCL10, CD8A, CD4, FOXP3, ICOS, CTLA4* | PMID: 25970248 |
| T effector signature | *GZMA, GZMB, PRF1, EOMES, IFNG, TNF, CXCL9, CXCL10, CD8A, CD4, FOXP3, ICOS, CTLA4* | PMID:25428504 |
| IFNG-related gene signature | *CD8A, CCL5, CD27, CD274, PDCD1LG2, CD276, CMKLR1, CXCL9, CXCR6, HLA-DQA1, HLA-DRB1, HLA-E, IDO1, LAG3, NKG7, PSMB10, STAT1, TIGIT* | PMID: 28650338 |
| Cytolytic activity signature | *GZMA, PRF1* | PMID:25594174 |

**Abbreviations**: MHC: major histocompatibility complex; IFNG: interferon-γ.

**Supplementary Table 8. Information about anti-CDK6 antibody used in IHC assay.**

| IHC | IHC antibody | Antibody source | Diluted | IHC scoring method |
| --- | --- | --- | --- | --- |
| CDK6 expression | Anti-CDK6 antibody (mRb) | Abcam ab124821 | 1:200 | IHC score |

**Abbreviations**: IHC: immunohistochemistry.
