# Supplementary material for: Integrative score based on CDK6, PD-L1 and TMB predicts response to platinum-based chemotherapy and PD-1/PD-L1 blockade in muscle-invasive bladder cancer
Source: Br J Cancer. 2024 Jan 11;130(5):852–60. doi: 10.1038/s41416-023-02572-9 (PMC10912081; doi:10.1038/s41416-023-02572-9)
Supplement: Supplementary file 2 — Supplementary Figures [file 41416_2023_2572_MOESM2_ESM.docx]

**Supplementary Figures**

**Supplementary Figure 1. Comprehensive information about patients enrolled in this study.**

**Supplementary Figure 2. Representative immunohistochemistry images of CDK6 expression.**

**Supplementary Figure 3. The predictive value of CDK6 on recurrence-free survival in patients with MIBC.**

**Supplementary Figure 4. Correlation of PD-L1 expression on tumour-infiltrating immune cells with CD274 mRNA expression in IMvigor210 cohort.**

**Supplementary Figure 5. Predictive value of integration of CDK6 with PD-L1 or TMB and integration of PD-L1 with TMB for ICI in MIBC patients.**

**Supplementary Figure 6. The predictive value of response score incorporating CDK6, PD-L1 and TMB on chemotherapy in MIBC.**

**Supplementary Figure 7. Correlation of response score with antigen presentation machinery in MIBC patients.**

**Supplementary Figure 8. Correlation of response score with tumour microenvironment subtype in TCGA cohort.**

**Supplementary Figure 9. Genomic landscape of MIBC stratified by response score in IMvigor210 cohort and UC-GENOME cohort.**


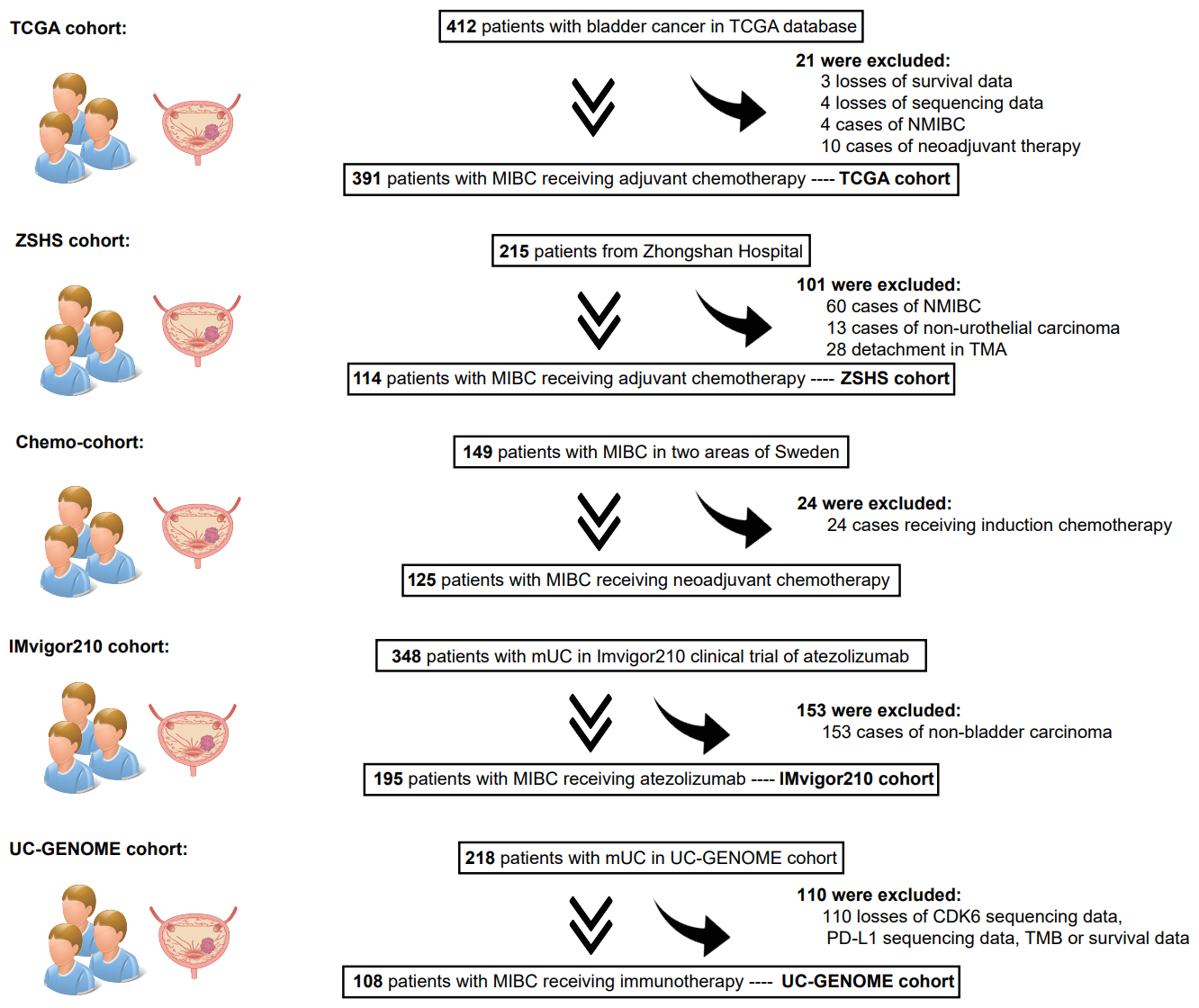


**Supplementary Figure 1. Comprehensive information about patients enrolled in this study.**


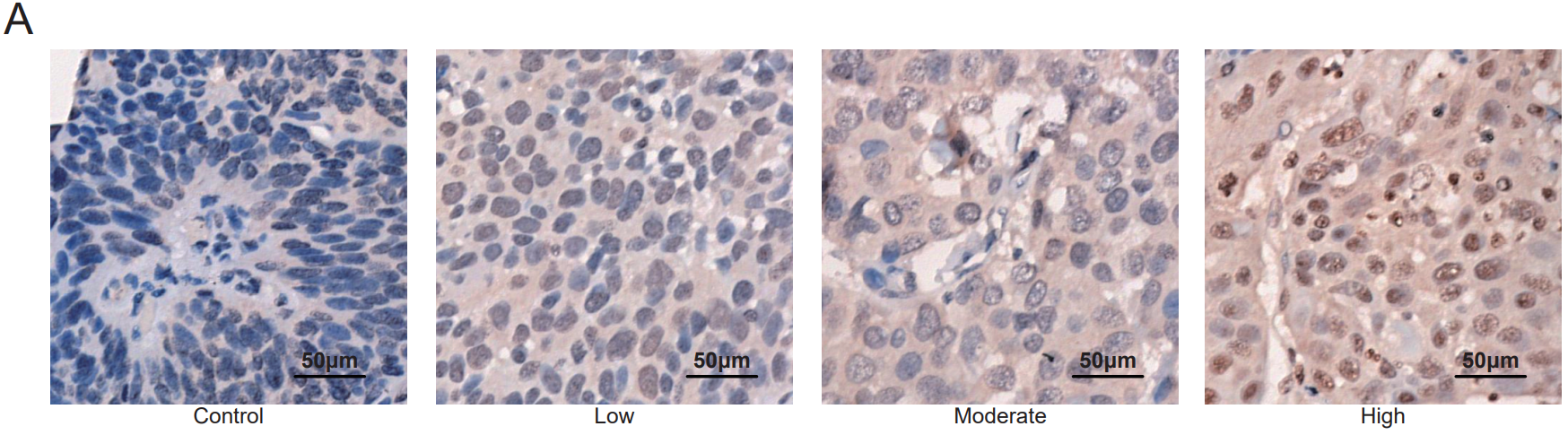


**Supplementary Figure 2. Representative immunohistochemistry images of CDK6 expression. Magnification: ×200.**


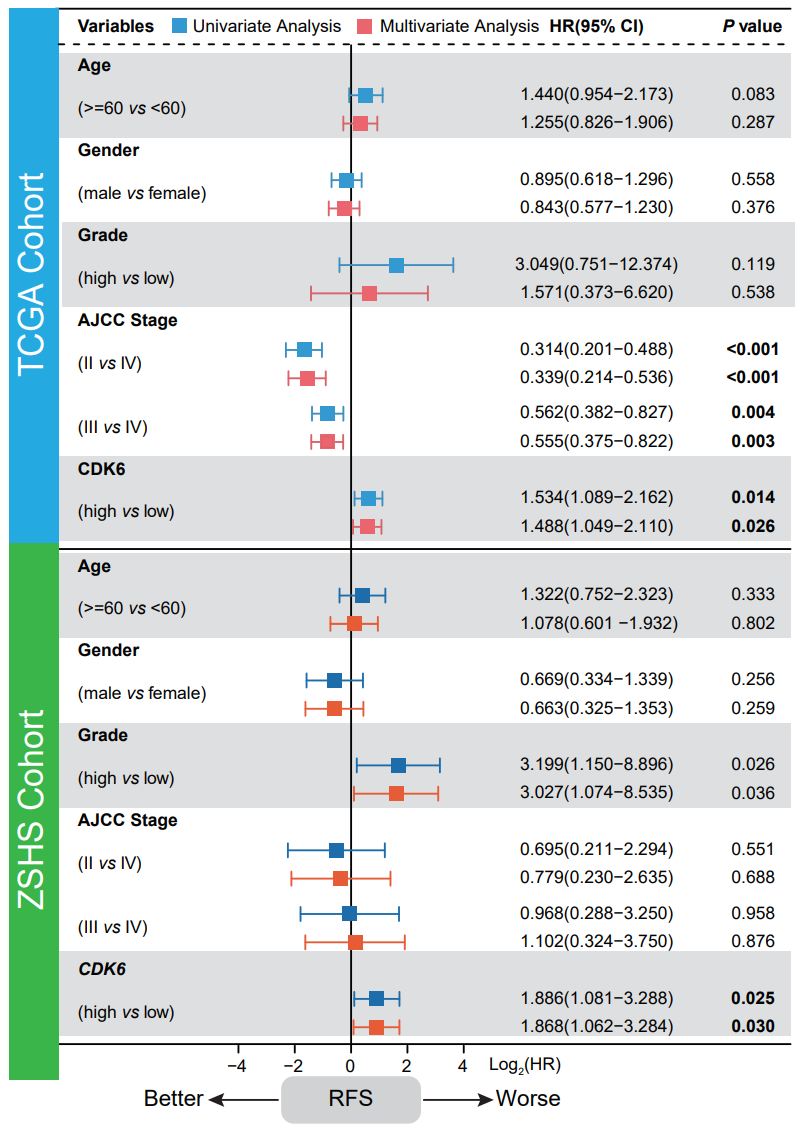


**Supplementary Figure 3. The predictive value of CDK6 on recurrence-free survival in patients with MIBC.** Univariate and Multivariate Cox regression of recurrence-free survival (RFS) based on CDK6 expression in TCGA cohort (n=391) and ZS cohort (n=114). Due to the limited number of patients at AJCC stage IV in ZSHS cohort, the cox regression outcomes of AJCC stage were not accurate enough. All reported *p* values were two sided. HR, hazard ratio; CI, confidence interval.


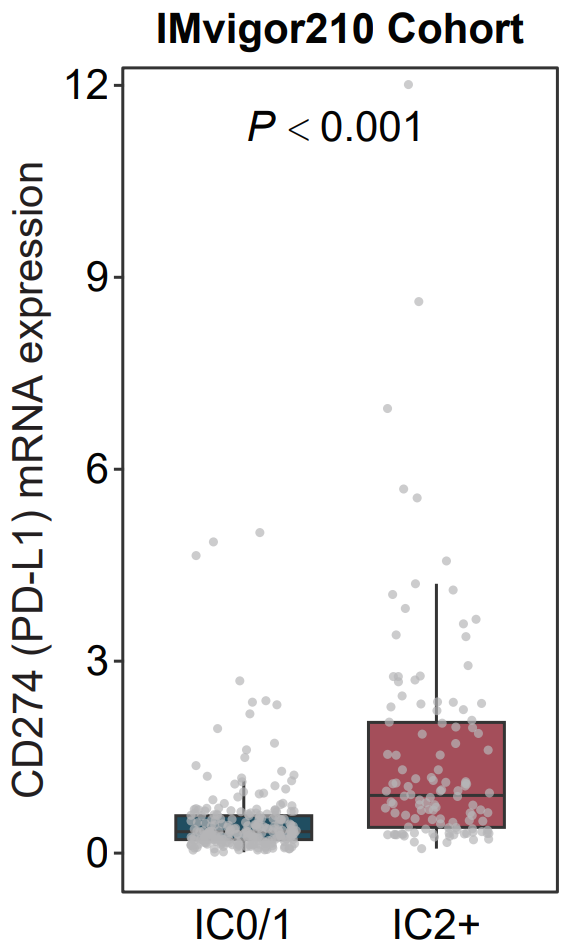


**Supplementary Figure 4. Correlation of PD-L1 expression on tumour-infiltrating immune cells with CD274 mRNA expression in IMvigor210 cohort.** Box plots illustrating the correlation of PD-L1 expression on tumour-infiltrating immune cells with CD274 mRNA expression in IMvigor210 cohort. Mann-Whitney test was applied. All reported *p* values were two sided. TME, IC: immune cell.


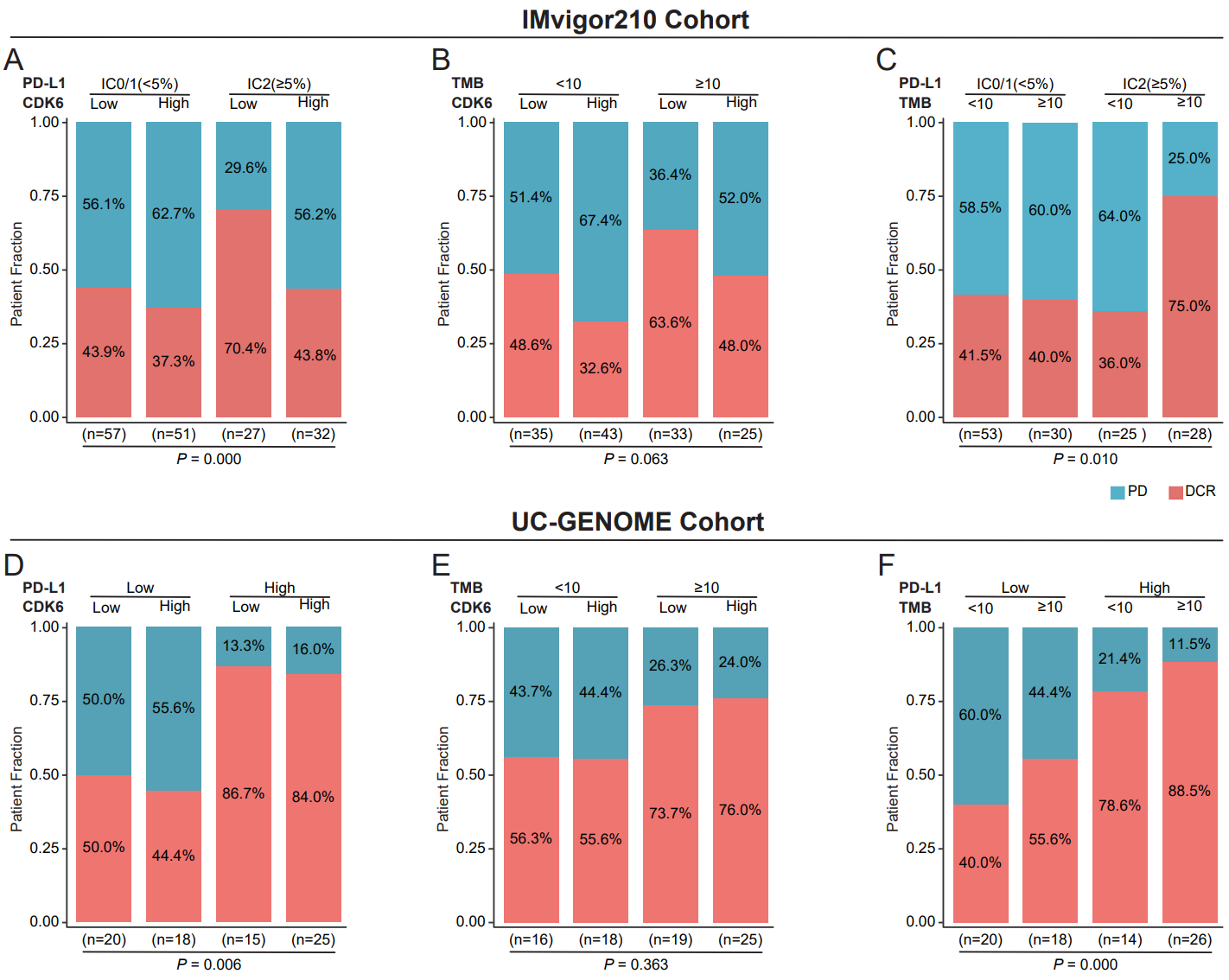


**Supplementary Figure 5. Predictive value of integration of CDK6 with PD-L1 or TMB and integration of PD-L1 with TMB for ICI in MIBC patients. (A-C)** Stacked bar plot demonstrated responsiveness to atezolizumab based on CDK6 expression combined with PD-L1 **(A)** or TMB **(B)** and integration of PD-L1 with TMB **(C)** in IMvigor210 cohort. **(D-F)** Stacked bar plot demonstrated responsiveness to ICI based on CDK6 expression combined with PD-L1 **(D)** or TMB **(E)** and integration of PD-L1 with TMB **(F)** in UC-GENOME cohort. χ^2^ test was applied. All reported p values were two sided.


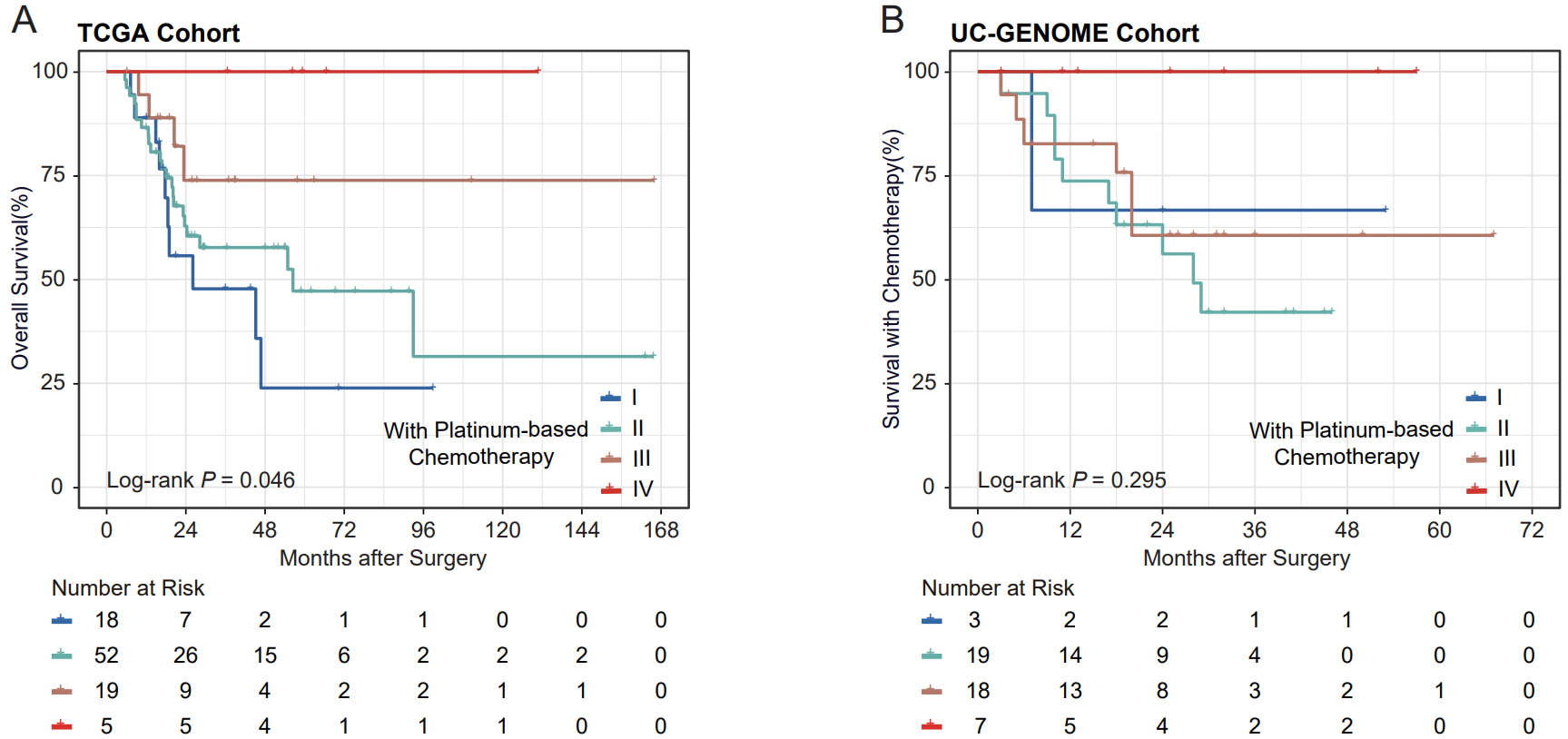


**Supplementary Figure 6. The predictive value of response score incorporating CDK6, PD-L1 and TMB on chemotherapy in MIBC. (A)** Kaplan-Meier curve of overall survival (OS) based on response score in patients who received platinum-based chemotherapy from TCGA cohort. **(B)** Kaplan-Meier curve of survival with chemotherapy based on response score in patients who received platinum-based chemotherapy from UC-GENOME cohort. Log-rank test was applied for Kaplan-Meier curves. All reported *p* values were two sided.


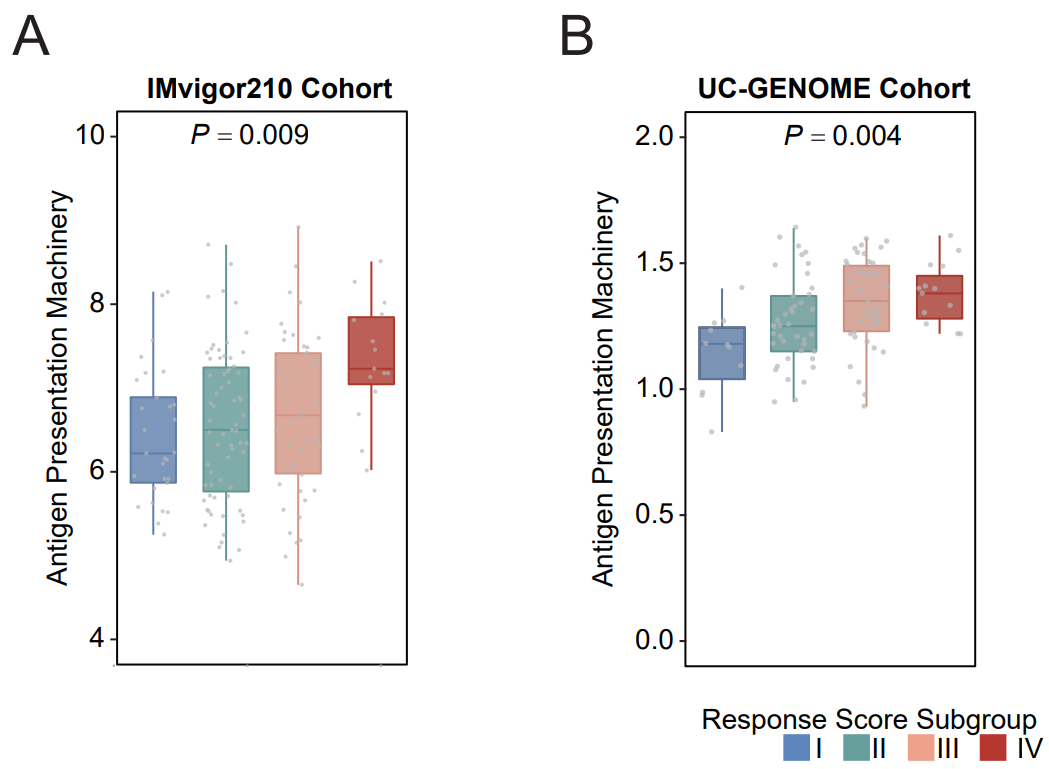


**Supplementary Figure 7. Correlation of response score with antigen presentation machinery in MIBC patients. (A, B)** Boxplots demonstrated the association between response score and antigen presentation machinery in IMvigor210 cohort **(A)** and UC-GENOME cohort **(B)**. Kruskal-Wallis test was applied. All reported *p* values were two sided.


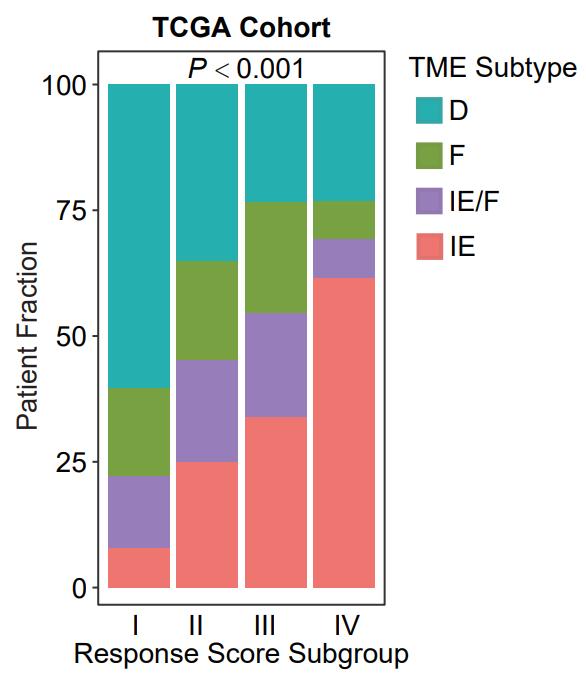


**Supplementary Figure 8. Correlation of response score with tumour microenvironment subtype in TCGA cohort.** Stacked bar chart illustrating the distribution of tumour microenvironment subtypes in response score subgroups in TCGA cohort. Chi-square test was applied. All reported *p* values were two sided. TME, tumour microenvironment; D, depleted; F, fibrotic; IE/F, immune-enriched, fibrotic; IE, immune-enriched, non-fibrotic.


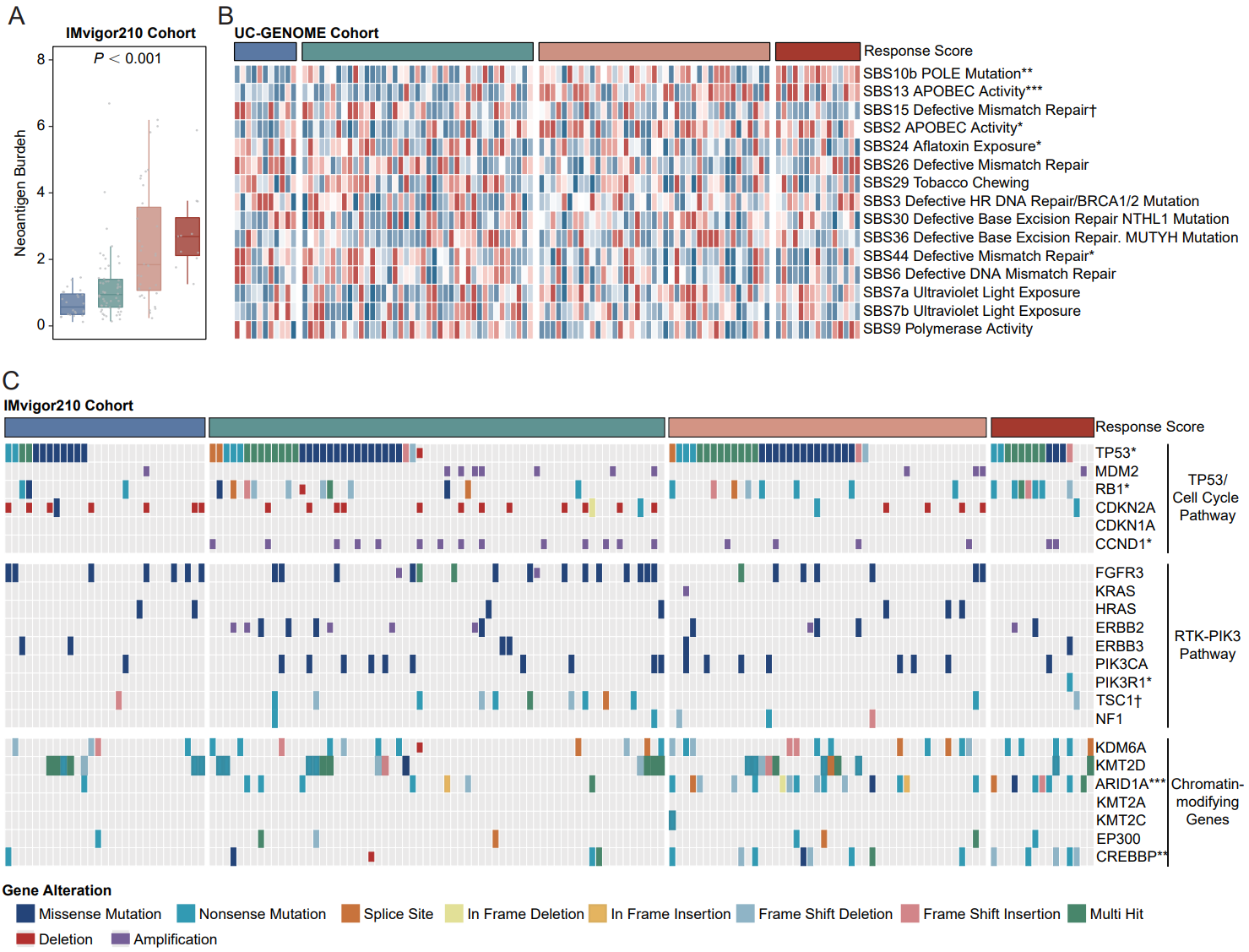


**Supplementary Figure 9. Genomic landscape of MIBC stratified by response score in IMvigor210 cohort and UC-GENOME cohort. (A)** Box plots showed the correlation of response score with neoantigen burden in IMvigor210 cohort. **(B)** Heatmap for the correlation between response score and mutation signature in UC-GENOME cohort. (C) Alteration landscape of genes from TP53/cell cycle pathway, RTK-PIK3 pathway and chromatin-modifying pathway in MIBC stratified by response score in IMvigor210 cohort. Kruskal-Wallis test, Chi-square test and Fisher’s exact test were applied. All reported *p* values were two sided. ^†^*P*<0.1, **p*<0.05, ***p*<0.01 and ****p*<0.001.
